# Database for High Throughput Screening Hits (dHITS): a simple tool to retrieve gene specific phenotypes from systematic screens done in yeast

**DOI:** 10.1002/yea.3312

**Published:** 2018-05-03

**Authors:** Silvia G. Chuartzman, Maya Schuldiner

**Affiliations:** ^1^ Department of Molecular Genetics, Weizmann Institute of Science Rehovot 7610001 Israel

**Keywords:** database, deletion library, dHITS, GFP library, phenotype, Saccharomyces cerevisiae, systematic screens, yeast libraries

## Abstract

In the last decade several collections of Saccharomyces cerevisiae yeast strains have been created. In these collections every gene is modified in a similar manner such as by a deletion or the addition of a protein tag. Such libraries have enabled a diversity of systematic screens, giving rise to large amounts of information regarding gene functions. However, often papers describing such screens focus on a single gene or a small set of genes and all other loci affecting the phenotype of choice (‘hits’) are only mentioned in tables that are provided as supplementary material and are often hard to retrieve or search. To help unify and make such data accessible, we have created a Database of High Throughput Screening Hits (dHITS). The dHITS database enables information to be obtained about screens in which genes of interest were found as well as the other genes that came up in that screen – all in a readily accessible and downloadable format. The ability to query large lists of genes at the same time provides a platform to easily analyse hits obtained from transcriptional analyses or other screens. We hope that this platform will serve as a tool to facilitate investigation of protein functions to the yeast community.

## 1 INTRODUCTION

The yeast Saccharomyces cerevisiae (from here on termed yeast) was the first eukaryote to have its full genome sequenced 20 years ago (Barrell et al., 1996). The availability of the full gene tally, with a finite number of only 6000 genes, drove a community effort to create arrayed, genome‐wide collections of genetically modified strains, colloquially termed libraries. In general, two types of libraries have since been created. The first are those intended to enable characterization of gene functions by altering gene sequence or levels and measuring/studying the effects of these manipulations. These include the whole‐genome deletion library (Giaever et al., 2002; Winzeler et al., 1999), various mutant libraries for essential genes [temperature sensitive alleles (Ben‐Aroya et al., 2008; Li et al., 2011); *TET‐off* promoters for repression of transcription (Mnaimneh et al., 2004); destabilization of mRNA for reduced expression (Breslow et al., 2008)] and an overexpression library (Sopko et al., 2006). The second type enabled the characterization of the proteins themselves by visualizing their localization, abundance or characteristics (such as protein–protein interactions) using various tags. Few examples for such libraries include the C terminus (′) tagged Green Fluorescence Protein (GFP) library (Huh et al., 2003) and the newly made N′ GFP and N′ Cherry libraries (Yofe et al., 2016).

The utilization of yeast for screening of gene functions has been practised since the 1980s (Bankaitis, Johnson, & Emr, 1986; Erdmann, Veenhuis, Mertens, & Kunau, 1989; Novick, Field, & Schekman, 1980) and was one of the drivers for making yeast a widely utilized model organism. However, until the creation of systematic, arrayed, libraries, screens were performed largely by random mutagenesis or pooled plasmid libraries, and hence were often not comprehensive or exhaustive and were nearly never quantitative. The creation of arrayed yeast libraries opened up a new approach to screening, providing a more systematic and quantitative capacity. For example, the first screens for the whole‐genome deletion libraries measured colony sizes in various media or stresses, giving a quantitative, statistically significant value for the ability of each strain to grow in a specific environment (Giaever et al., 2002). With the advent of more sophisticated robotic setups that enabled integration of additional genetic traits into libraries (Cohen & Schuldiner, 2011; Tong et al., 2001) and measurement of more complex phenotypes using microscopy as readouts, the quantity of screens and their information content grew dramatically.

**Figure 1:**
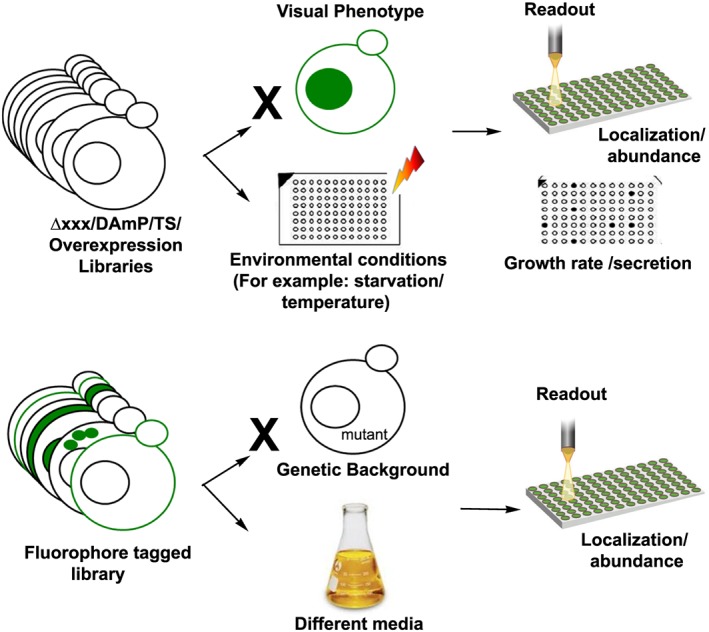
Schematic representation of the two types of screens that are represented in the dHITS database

Over the years it is becoming apparent that most yeast screens fall into one of two categories (Figure 1):

1. An altered‐expression library (e.g. the deletion library, the hypomorphic allele library and the overexpression library), is used to search for phenotypes for a given gene. Such phenotypes can be diverse (a few examples include growth rate, drug resistance, secretion and cellular localization/abundance of a query protein). Such screens can often be done with manual approaches and hence are more readily performed.
2. A fluorophore‐tagged library (e.g. the GFP library) is used to identify changes in localization or abundance of all proteins under a specific genetic background (e.g. deletion of a gene) or growth condition (e.g media). Screens such as this require a high content screening setup and are therefore less prevalent.

To date, tens of such screens have already been performed and the wealth of information that they provide has been extremely helpful in characterizing protein functions. However, often finding information about such screens is not a trivial task. One reason for this is that ‘hit’ lists from screens often only show up in supplementary materials of publications and have a variety of different layouts and terminologies. Since such lists do not appear in the abstract or main text, they often do not come up during literature searches and can be missed by someone interested in the function of a specific gene or group of genes. By annotating all the information from such screens, the *Saccharomyces genome database* (SGD) (SGD, 2018) has created an invaluable resources available to the yeast community. While the SGD continues to be the most comprehensive and accurately curated yeast screen database, it is not simple to use it to compare hit lists from several different screens or to search for genes with a similar hit pattern. To make querying this information more accessible and direct, we have created a new platform that concentrates screens from the two above types in a single, easy to use, database: dHITS (Database for High Throughput Screen Hits; https://www.dhitsmayalab.tk/firstPage.php (direct entry) http://mayaschuldiner.wixsite.com/schuldinerlab/dhits (alternative address).

**Figure 2:**
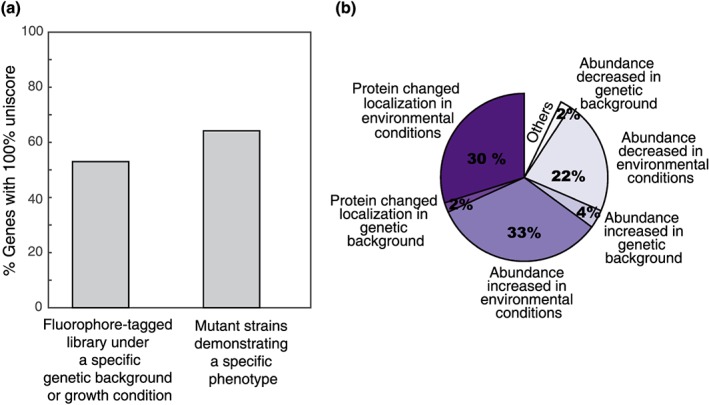
Statistical analysis of strains curated in dHITS. (a) Bar graph of the number of strains that have a uniscore of 100% from each screen category. (b) Pie chart representing the diversity of phenotypes observed with the fluorophore tagged strains [Colour figure can be viewed at http://wileyonlinelibrary.com]

The dHITS database has several unique characteristics to optimize its utilization by the yeast community:

1. *Querying lists of genes* – the dHITS database is built to enable querying large groups of genes for their appearance in screens. This is especially helpful when researchers have lists of genes from deep‐sequencing, micro‐array or screening efforts and are looking for possible connections to their process of interest. In essence, dHITS enables easy discovery of additional phenotypes for a list of genes that will help give functional predictions to a gene of choice.
2. *Curation –* the dHITS database is unique in that each high‐throughput screen that is represented has been curated to enable easy understanding of both the screen itself and the phenotypes observed. In addition, hit lists are given in an organized, consistent and easy to download format.
3. *Uniscore –* one of the distinctive features of the dHITS database is an internal calculation of uniqueness that we term Uniscore. Uniscore gives a numerical value to how many times a gene has appeared in screens. This parameter can be used to differentiate non‐specific, pleiotropic effects of a given deletion (low Uniscore) vs. highly specific effects of a given gene on a process of choice (high Uniscore). For example, we find that 50% of fluorophore tagged proteins have never been altered in expression or localization in any of the curated screens (Figure 2a). Similarly, over 60% of mutant strains have never displayed a phenotype in our curated screens (Figure 2a).
4. *Accessibility –*since dHITS was built to enable easy access to all yeast researchers to screening data, one of the main features is the ability to easily download all primary hit lists for each screen or all the screens for a given gene.

We hope that the unique capabilities of dHITS and the concentration of systematic screens into one, searchable database that will continue to grow and evolve as new screens continue to be published will enable *in silico* exploration of gene functions.

## 2 STEP‐BY‐STEP DESCRIPTION OF HOW TO USE ‘dHITS’

1. To search for phenotypes of a single gene or a list of genes, enter the dHITS database at: https://www.dhitsmayalab.tk/firstPage.php (direct entry) http://mayaschuldiner.wixsite.com/schuldinerlab/dhits (alternative address). In the homepage you will be prompted to choose the type of information you are seeking: (a) phenotypes resulting from alteration in gene functions (Figure 1, top choice in database); or (b) phenotypes resulting in changes in abundance/localization of proteins of choice (Figure 1, bottom choice in database). Only one type of data can be retrieved in each run.
2. Once a choice is made, users are automatically transferred to the next stage where they must enter a list of gene names. To maximize ease of use, such a list can be given as systematic gene names (such as *YGL020C*), standard names (*GET1*) or mixed, all in a manner that is insensitive to case. Lists can be copied and pasted directly from Excel files and manual entries must be separated by a paragraph mark. The requirements for correct entry as well as an example for a potential query are given. Once the list has been created, pressing the ‘Submit’ button will retrieve the results.
3. The results page is headed by the number of screens currently available for this type of data and which were mined for this analysis. We hope that with time this number will increase as more such screens become available and as we annotate more of them into dHITS.

This is followed by a table that includes several columns:

1. List of genes with their systematic names.
2. List of standard names.
3. Description of each gene as it appears in SGD (updated every six months).
4. Uniscore – a calculation of the number of screens out of the total number of screens analysed where the gene of choice demonstrated a phenotype. A Uniscore of 100% means that this gene never came up as a hit in previous screens and hence is unique.
5. A numbered list of the screens in which each gene was a hit. Each screen is a hyperlink to an explanation of the experimental setup for that screen. Entering the hyperlink also enables downloading of all of the hits for that specific screen.
6. A numbered list of the phenotypes related to each screen.
7. A link to Pubmed with the reference for each given screen in order to facilitate the retrieval of information on any screen for further use.

The table with all of the information can be downloaded by pressing the ‘Save as Csv file’ button at the bottom of each table.

In addition, any queries regarding utilization of the database or interest in uploading screen hits can be directed to us via the ‘Contact us’ button present in the home page. Finally, there is an option to view all manuscripts included in the database and directly download all of the hit lists by pressing the ‘List of papers included in this database’ button in the home page.

## 3 ‘dHITS' DATABASE CONSTRUCTION’

In order to collect as many high‐throughput screens into the dHITS database we first uploaded all of the published screens from our laboratory to date. This includes screens of either the deletion/DAmP library or of the GFP library under a variety of genetic and environmental conditions (For breakdown of the various phenotypes of the GFP tagged strains see Figure 2b). As a next step we mined the literature for similar high‐throughput screens, downloaded the tables describing the hits of these screens and unified data presentation and terminology with our data style. All of the papers that we have currently curated and the numbers of hits that came up in their respective screens are provided in Table 1.

**Table 1:** All papers currently represented in dHITS and the quantity of hits arising from each screen

Title of Manuscript	Reference	Number of hits in screen
Genetic basis of mitochondrial function and morphology in Saccharomyces cerevisiae	Dimmer et al. (2002)	401
Genomic screen for vacuolar protein sorting genes in *Saccharomyces cerevisiae*	Bonangelino, Chavez, and Bonifacino (2002)	146
A genome‐wide visual screen reveals a role for sphingolipids and ergosterol in cell surface delivery in yeast	Proszynski et al. (2005)	22
Role of essential genes in mitochondrial morphogenesis in *Saccharomyces cerevisiae*	Altmann and Westermann (2005)	119
A proteomic screen reveals SCFGrr1 targets that regulate the glycolytic‐gluconeogenic switch	Benanti, Cheung, Brady, and Toczyski (2007)	163
The lipodystrophy protein seipin is found at endoplasmic reticulum lipid droplet junctions and is important for droplet morphology	Szymanski et al. (2007)	59
Global screening of genes essential for growth in high‐pressure and cold environments: searching for basic adaptive strategies using a yeast deletion library	Abe and Minegishi (2008)	80
Comprehensive phenotypic analysis for identification of genes affecting growth under ethanol stress in *Saccharomyces cerevisiae*	Yoshikawa et al. (2009)	446
Genome wide analysis reveals novel pathways affecting endoplasmic reticulum homeostasis, protein modification and quality control	Copic et al. (2009)	72
Imaging‐based live cell yeast screen identifies novel factors involved in peroxisome assembly	Wolinski et al. (2009)	31
The Rpd3L HDAC complex is essential for the heat stress response in yeast	Ruiz‐Roig, Vieitez, Posas, and de Nadal (2010)	276
Ergosterol content specifies targeting of tail‐anchored proteins to mitochondrial outer membranes	Krumpe et al. (2012)	1
Interactions of subunit CCT3 in the yeast chaperonin CCT/TRiC with Q/N‐rich proteins revealed by high‐throughput microscopy analysis	Nadler‐Holly et al. (2012)	64
Identification of genes affecting vacuole membrane fragmentation in *Saccharomyces cerevisiae*	Michaillat and Mayer (2013)	276
Formation and dissociation of proteasome storage granules are regulated by cytosolic pH	Peters, Hazan, Breker, Schuldiner, and Ben‐Aroya (2013)	11
A novel single‐cell screening platform reveals proteome plasticity during yeast stress responses (C′ GFP in DTT)	Breker, Gymrek, and Schuldiner (2013)	421
A novel single‐cell screening platform reveals proteome plasticity during yeast stress responses (C′ GFP in starvation)	Breker et al. (2013)	885
Genome‐wide single‐cell‐level screen for protein abundance and localization changes in response to DNA damage in S. cerevisiae	Mazumder, Pesudo, McRee, Bathe, and Samson (2013)	1697
The role of Djp1 in import of the mitochondrial protein Mim1 demonstrates specificity between a cochaperone and its substrate protein	Papic et al. (2013)	10
A defect in the RNA‐processing protein HNRPDL causes limb‐girdle muscular dystrophy 1G (LGMD1G)	Vieira et al. (2014)	17
A dynamic interface between vacuoles and mitochondria in yeast	Elbaz‐Alon, Rosenfeld‐Gur et al. (2014)	118
A functional, genome‐wide evaluation of liposensitive yeast identifies the ‘ARE2 required for viability’ (ARV1) gene product as a major component of eukaryotic fatty acid resistance	Ruggles et al. (2014)	143
Genome‐wide analysis of *Saccharomyces cerevisiae* identifies cellular processes affecting intracellular aggregation of Alzheimer's amyloid‐beta42: importance of lipid homeostasis	Nair, Traini, Dawes, and Perrone (2014)	332
Peroxisomes are juxtaposed to strategic sites on mitochondria	Cohen et al. (2014)	55
The yeast oligopeptide transporter Opt2 is localized to peroxisomes and affects glutathione redox homeostasis	Elbaz‐Alon, Morgan et al. (2014)	22
The yeast ER‐intramembrane protease Ypf1 refines nutrient sensing by regulating transporter abundance	Avci et al. (2014)	50
Yeast phospholipid biosynthesis is linked to mRNA localization	Hermesh et al. (2014)	14
Genome‐wide screen uncovers novel pathways for tRNA processing and nuclear‐cytoplasmic dynamics	Wu, Bao, Chatterjee, Wan, and Hopper (2015)	172
Genome‐wide screens in *Saccharomyces cerevisiae* highlight a role for cardiolipin in biogenesis of mitochondrial outer membrane multispan proteins	Sauerwald et al. (2015)	144
Lipid droplets are essential for efficient clearance of cytosolic inclusion bodies	Moldavski et al. (2015)	59
Starvation‐dependent regulation of Golgi quality control links the TOR signaling and vacuolar protein sorting pathways	Dobzinski, Chuartzman, Kama, Schuldiner, and Gerst (2015)	25
An unrecognized function for COPII components in recruiting the viral replication protein BMV 1a to the perinuclear ER	Li et al. (2016)	17
Molecular insight into arsenic toxicity via the genome‐wide deletion mutant screening of *Saccharomyces cerevisiae*	Johnson et al. (2016)	114
Water‐transfer slows aging in *Saccharomyces cerevisiae*	Cohen et al. (2016)	424
Characterization of proteome dynamics in oleate reveals a novel peroxisome targeting receptor (C′ GFP in oleate)	Yifrach et al. (2016)	461
Characterization of proteome dynamics in oleate reveals a novel peroxisome targeting receptor (N′ GFP in oleate)	Yifrach et al. (2016)	718
The Protease Ste24 clears clogged translocons	Ast, Michaelis, and Schuldiner (2016)	106
The SND proteins constitute an alternative targeting route to the endoplasmic reticulum	Aviram et al. (2016)	91
Combining deep sequencing, proteomics, phosphoproteomics, and functional screens to discover novel regulators of sphingolipid homeostasis	Lebesgue et al. (2017)	569
Pex35 is a regulator of peroxisome abundance	Yofe et al. (2017)	43
Cellular consequences of diminished protein *O*‐mannosyltransferase activity in bakers yeast	Zatorska et al. (2017)	21
Iron affects Ire1 clustering propensity and the amplitude of endoplasmic reticulum stress signaling	Cohen et al. (2017)	153
A pathway of targeted autophagy is induced by DNA damage in budding yeast	Eapen et al. (2017)	21
Identification of seipin‐linked factors that act as determinants of a lipid droplet subpopulation (Pdr16‐Cherry)	Eisenberg‐Bord et al. (2018)	49
Identification of seipin‐linked factors that act as determinants of a lipid droplet subpopulation (Galp‐GFP‐LDO45)	Eisenberg‐Bord et al. (2018)	46

Importantly there were several types of data that we did not integrate into the dHITS database. First, we did not integrate genetic and physical interaction scores as there are numerous, well‐annotated websites that enable mining such data. We also did not include complex phenotypes such as lipidomic, ionomic or metabolomic datasets. Finally, we did not include huge datasets from chemogenomic profiling of the deletion library (Dudley, Janse, Tanay, Shamir, & Church, 2005; Hillenmeyer et al., 2008) as these have their own, easily mineable interface. This is because we wanted to focus the dHITS database on individual screens that had specific, single, phenotypic outcomes.

Our literature analysis and curation may not have been comprehensive or exhaustive and new screens are continuously being published, and hence we encourage any laboratory interested in uploading data from their screen to contact us.

## 4 SUMMARY

We here describe a new database that we have created to organize and categorize two types of whole‐genome screens in yeast – those querying the phenotypic consequence of altering a single gene and those measuring changes in protein abundance or localization of a protein of interest. We hope that this database will become a new platform for integrating hits from future screens as they become available. By pooling information from a multitude of laboratories and approaches into a single, unified, searchable database we hope to provide a new, powerful, tool for investigation of protein functions in the most studied, yet still little understood, model eukaryote, the yeast *Saccharomyces cerevisiae*.
